# Dihydroceramides Derived from Bacteroidetes Species Sensitize TRPV1 Channels

**DOI:** 10.3390/ijms24010877

**Published:** 2023-01-03

**Authors:** Nora Ludwig, Isaac S. Demaree, Chiaki Yamada, Amilia Nusbaum, Frank C. Nichols, Fletcher A. White, Alexandru Movila, Alexander G. Obukhov

**Affiliations:** 1 Department of Anatomy, Cell Biology & Physiology, Indiana University School of Medicine, Indianapolis, IN 46202, USA; 2 Department of Biomedical Sciences and Comprehensive Care, Indiana University School of Dentistry, Indianapolis, IN 46202, USA; 3 Indiana Center for Musculoskeletal Health, Indiana University School of Medicine, Indianapolis, IN 46202, USA; 4 Department of Oral Health and Diagnostic Sciences, University of Connecticut School of Dental Medicine, Farmington, CT 06030, USA; 5 Stark Neurosciences Research Institute, Indiana University School of Medicine, Indianapolis, IN 46202, USA; 6 Department of Anesthesia, Indiana University School of Medicine, Indianapolis, IN 46202, USA; 7 Richard L. Roudebush Veterans Medical Center, Indianapolis, IN 46202, USA

**Keywords:** dihydroceramides, *Bacteroides* spp., TRPV1 channels, Ca^2+^ influx

## Abstract

Bacterial colonization of open wounds is common, and patients with infected wounds often report significantly elevated pain sensitivity at the wound site. Transient Receptor Potential Vanilloid Type 1 (TRPV1) channels are known to play an important role in pain signaling and may be sensitized under pro-inflammatory conditions. Bacterial membrane components, such as phosphoethanolamine dihydroceramide (PEDHC), phosphoglycerol dihydroceramide (PGDHC), and lipopolysaccharide (LPS), are released in the environment from the Gram-negative bacteria of the Bacteroidetes species colonizing the infected wounds. Here, we used intracellular calcium imaging and patch-clamp electrophysiology approaches to determine whether bacterially derived PEDHC, PGDHC, or LPS can modulate the activity of the TRPV1 channels heterologously expressed in HEK cells. We found that PEDHC and PGDHC can sensitize TRPV1 in a concentration-dependent manner, whereas LPS treatment does not significantly affect TRPV1 activity in HEK cells. We propose that sensitization of TRPV1 channels by Bacteroidetes-derived dihydroceramides may at least in part underlie the increased pain sensitivity associated with wound infections.

## 1. Introduction

TRPV1 channels are known to play a critical role in pain signaling and are highly expressed on the nociceptive nerve endings innervating the skin and tooth pulp [1,2]. TRPV1 channels are permeable to Ca^2+^ and can be activated by heat, acid, capsaicin, and several endogenous mediators [1]. Remarkably, TRPV1 can be sensitized and exhibits enhanced response to its agonists (capsaicin and heat) under proinflammatory conditions and in the presence of prostaglandins, histamine, or H^+^ [1].

Clinical evidence indicates that peripheral TRPV1 nociceptive nerves are important for wound-healing processes [3], but sensitization of TRPV1 can contribute to the hyperalgesia observed in patients with infected wounds. Indeed, patients with bacterially infected wounds present with increased pain compared to those patients who have uninfected wounds [4,5]. *Bacteroidetes* species, Gram-negative and anaerobic bacteria [6], are known to colonize wounds [7,8,9]. Specifically, *Bacteroidetes fragilis* was identified in infected postsurgical sites [7,8,9], and *Bacteroidetes pyogenes* was found in polymicrobial animal-bite wounds and diabetic foot infections [10]. *Bacteroidetes* species shed lipopolysaccharides (LPS) into the environment, and LPS may be responsible for the hyperalgesia observed in patients with infected wounds [11,12], exacerbating pain by sensitizing neuronal TRPV1 via the Toll-like receptor 4 (TLR4) axis [13]. However, LPS is not the only bacterial component shed by *Bacteroidetes* species. Recently, a novel group of sphingolipids was identified in gut and oral *Bacteroides* species. This new group includes phosphoethanolamine dihydroceramide (PEDHC) and phosphoglycerol dihydroceramide (PGDHC), which are produced and shed by *Bacteroides* species in relatively large amounts [3,14,15,16].

A growing body of evidence suggests that the cell-permeable dihydroceramides may promote inflammation independent of the canonical signaling elicited by TLR4 [3,14,17]. These bacterially derived molecules may also act more directly on the TRPV1 protein. In this study, we utilized intracellular calcium imaging and patch-clamp electrophysiology approaches to determine if PEDHC, PGDHC, or LPS can sensitize human TRPV1 channels to capsaicin activation in the HEK heterologous expression model lacking the TLR4 receptor.

## 2. Results

**Figure 1:**
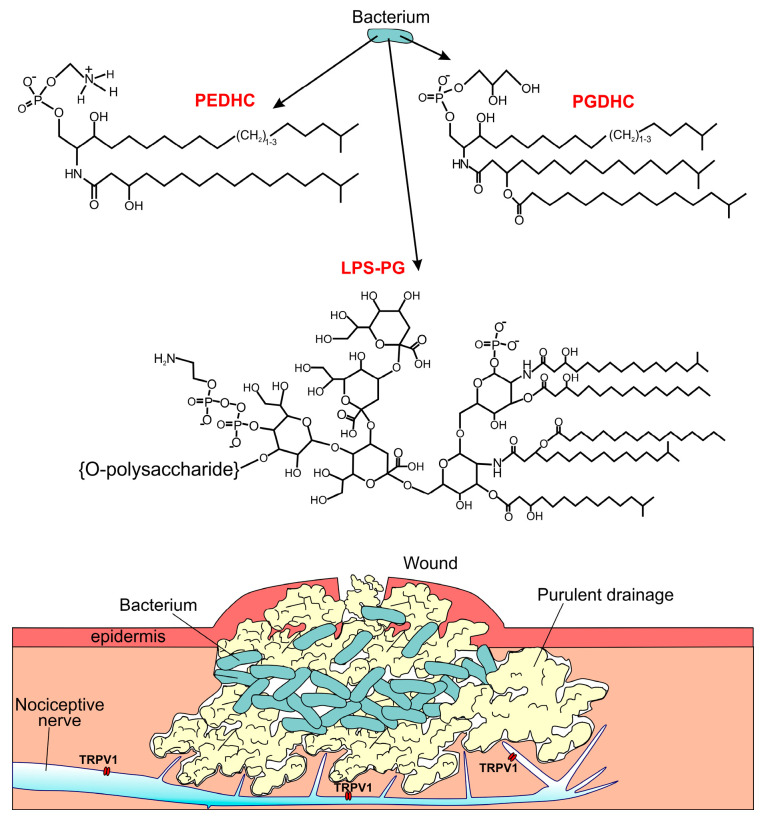
*Bacteroidetes spp.* release several membrane components into the environment, such as phosphoethanolamine dihydroceramide (PEDHC), phosphoglycerol dihydroceramide (PGDHC), and lipopolysaccharides (LPS). The upper panel of the figure shows molecular structures of the named components (LPS from *Porphyromonas gingivalis,* LPS-PG, is shown; [18]). The lower panel depicts a drawing of bacterially infected wound.

*Bacteroidetes* spp. release LPS, PEDHC, and PGDHC into the environment (Figure 1). Here, we used intracellular Fura-2-based Ca^2+^ imaging to determine whether TRPV1 activity is influenced by LPS, PEDHC, or PGDHC in a TLR4-independent manner.

**Figure 2:**
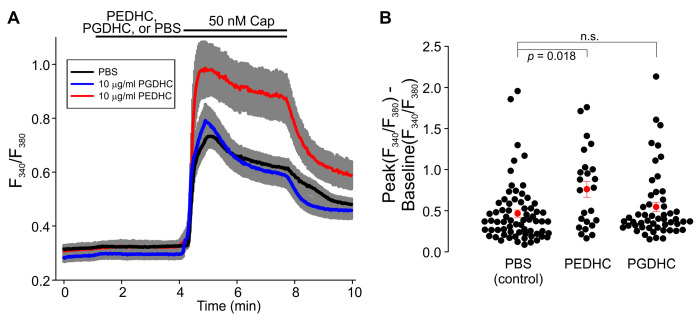
The effect of high concentration of dihydroceramides (10 μg/mL) on capsaicin-induced intracellular Ca^2+^ increases measured using Fura-2 fluorescence imaging. (**A**) F_340_/F_380_ ratio increases were greater in cells pretreated with PEDHC (10 μg/mL) compared to cells pretreated with PBS or PGDHC (PBS, *n* = 68; PEDHC, *n* = 24; PGDHC, *n* = 52). The gray area indicates ± SEM. The solid lines are the means of F_340_/F_380_ ratio changes over time recorded in each individual cell. The horizontal bars denote the times when the indicated compounds were present in the bath. (**B**) Summary of the data presented in (**A**). The differences between the peak values of capsaicin-induced Fura-2 F_340_/F_380_ ratio increases and the F_340_/F_380_ ratio at the baseline were determined in each tested TRPV1-HEK cell. The filled red circles represent the mean difference values for each experimental group, and the vertical red lines are the error bars (SEM). The Kruskal–Wallis one-way analysis of variance (ANOVA) on ranks test, followed by the Dunn’s post hoc multiple comparisons versus control group test, was used to determine whether there is a significant difference between the tested groups. A statistically significant difference was observed only between the PEDHC and PBS groups at this concentration (*p* = 0.018).

**Figure 3:**
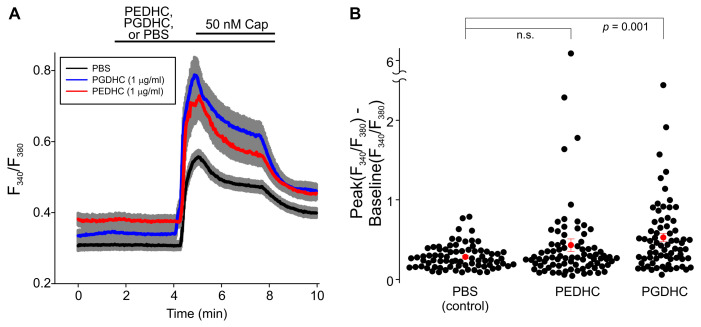
The effect of intermediate concentration of dihydroceramides (1 μg/mL) on capsaicin-induced intracellular Ca^2+^ increases measured using Fura-2 fluorescence imaging. (**A**) F_340_/F_380_ ratio increases were greater in cells pretreated with PGDHC (1 μg/mL) compared to cells pretreated with PBS or PEDHC (1 μg/mL) (PBS, *n* = 76; PEDHC, *n* = 83; PGDHC, *n* = 70). The gray area indicates ± SEM. The solid lines are the means of F_340_/F_380_ ratio changes over time recorded in each individual cell. The horizontal bars denote the times when the indicated compounds were present in the bath. (**B**) Summary of the data presented in (**A**). The differences between the peak values of capsaicin-induced F_340_/F_380_ ratio increases and the F_340_/F_380_ ratio at the baseline were determined in each tested TRPV1-HEK cell. The filled red circles represent the mean difference values for each experimental group, and the vertical red lines are the error bars (SEM). The Kruskal–Wallis one-way ANOVA on ranks test, followed by the Dunn’s post hoc multiple comparisons versus control group test, was used to determine whether there is a significant difference between the tested groups. A statistically significant difference was observed only between the PGDHC and PBS groups at this concentration (*p* = 0.001).

**Figure 4:**
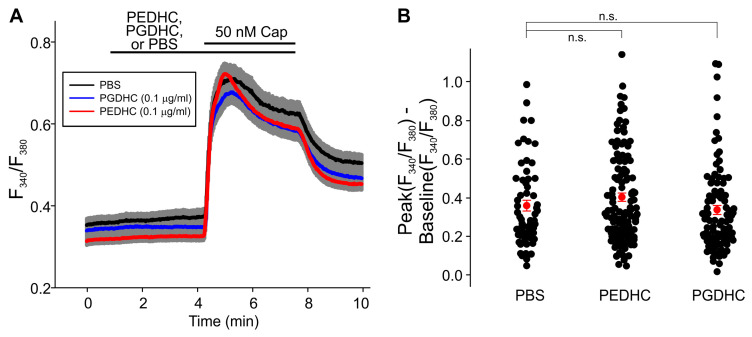
Low concentration of neither PGDHC (0.1 μg/mL) nor PEDHC (0.1 μg/mL) altered capsaicin-induced intracellular Ca^2+^ increases measured using Fura-2 fluorescence imaging. (**A**) F_340_/F_380_ ratio increases were not different in cells pretreated with PGDHC (0.1 μg/mL) or PEDHC (0.1 μg/mL) compared to cells pretreated with PBS (PBS, *n* = 60; PEDHC, *n* = 116; PGDHC, *n* = 94). The gray area indicates ± SEM. The solid lines are the means of F_340_/F_380_ ratio changes over time recorded in each individual cell. The horizontal bars denote the times when the indicated compounds were present in the bath. (**B**) Summary of the data presented in (**A**). The differences between the peak values of capsaicin-induced F_340_/F_380_ ratio increases and the F_340_/F_380_ ratio at the baseline were determined in each tested TRPV1-HEK cell. The filled red circles represent the mean difference values for each experimental group, and the vertical red lines are the error bars (SEM). The Kruskal–Wallis one-way ANOVA on ranks test was used to determine whether there is a significant difference between the tested groups. No statistically significant difference was observed between the tested groups at this concentration (*p* = 0.072).

Neither LPS (10 μg/mL), nor PEDHC (10 μg/mL), nor PGDHC (10 μg/mL) elicited Ca^2+^ influx in TRPV1-HEK cells. Pretreatment with PGDHC (10 μg/mL) also did not affect capsaicin-induced Ca^2+^ influx in TRPV1-HEK cells (Figure 2A). Conversely, pretreatment with PEDHC (10 μg/mL) significantly potentiated capsaicin-induced Ca^2+^ influx in TRPV1-HEK cells compared to PBS-pretreated TRPV1-HEK cells (Figure 2B). However, when a 1 µg/mL concentration of these two dihydroceramides was tested, we found that PGDHC but not PEDHC significantly potentiated capsaicin-induced Ca^2+^ influx, likely due to TRPV1 sensitization (Figure 3). When a 0.1 µg/mL concentration of these two dihydroceramides was tested, neither PEDHC nor PGDHC significantly affected capsaicin-induced currents in TRPV1-HEK cells (Figure 4). Thus, it appears that the effects of pretreatment with PEDHC or PGDHC were concentration-dependent.

**Figure 5:**
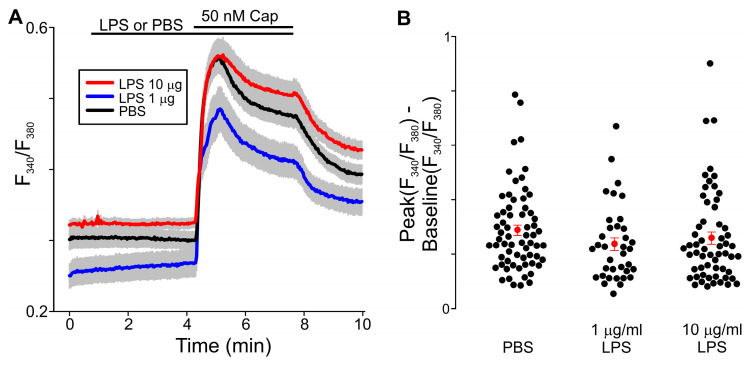
LPS-PG (1 μg/mL and 10 μg/mL) pretreatment did not affect capsaicin-induced intracellular Ca^2+^ increases measured using Fura-2 fluorescence imaging in TRPV1-HEK cells. (**A**) F_340_/F_380_ ratio increases were not significantly different in cells pretreated with LPS-PG (1 μg/mL) or LPS-PG (10 μg/mL) compared to cells pretreated with vehicle control (PBS, *n* = 65; LPS-PG 10 μg/mL, *n* = 58; LPS-PG 1 μg/mL, *n* = 36). The gray area indicates ± SEM. The solid lines are the means of F_340_/F_380_ ratio changes over time recorded in each individual cell. The horizontal bars denote the times when the indicated compounds were present in the bath. (**B**) Summary of the data presented in (**A**). The differences between the peak values of capsaicin-induced F_340_/F_380_ ratio increases and the F_340_/F_380_ ratio at the baseline were determined in each tested TRPV1-HEK cell. The filled red circles represent the mean difference values for each experimental group, and the vertical red lines are the error bars (SEM). No statistically significant difference was observed between each LPS-PG group and the PBS group (Kruskal–Wallis one-way ANOVA on ranks test, *p* = 0.111).

We next set out to determine the effect of LPS-PG on the capsaicin-induced intracellular Ca^2+^ increases in TRPV1-HEK cells. We tested two concentrations of LPS-PG, namely 1 µg/mL and 10 µg/mL. Our experiments revealed that LPS-PG pretreatment did not significantly alter capsaicin-induced intracellular Ca^2+^ increases in TRPV1-HEK cells compared to PBS-pretreated TRPV1-HEK cells at all tested concentrations (Figure 5).

**Figure 6:**
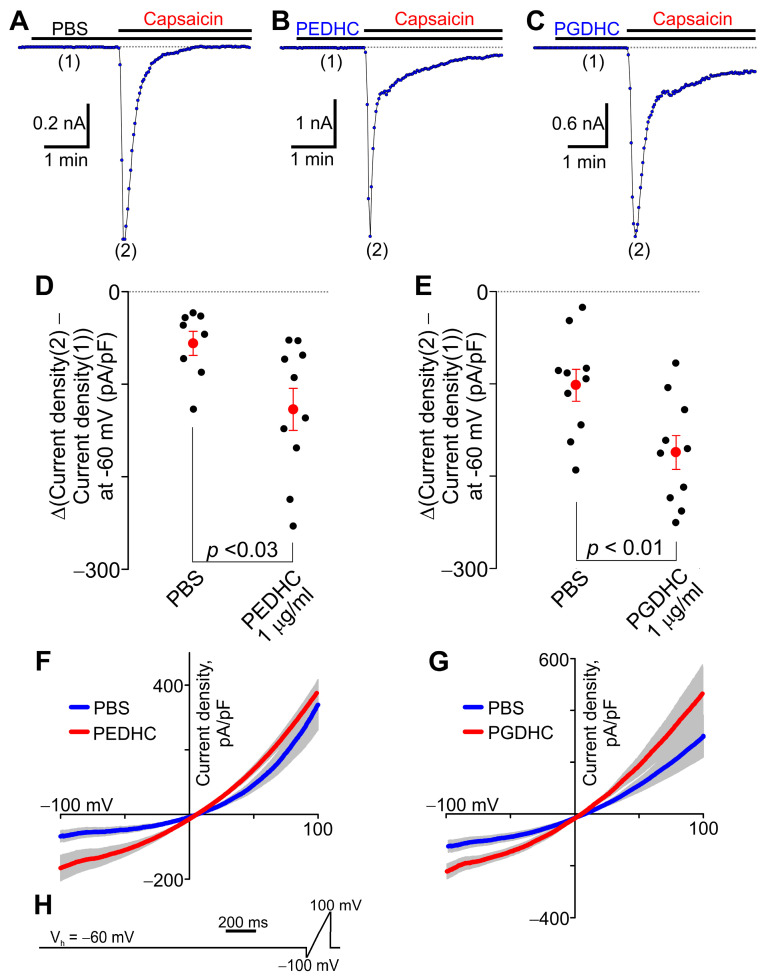
Effect of dihydroceramides on capsaicin-activated currents in TRPV1-HEK cells. The recordings were obtained using the whole-cell voltage-clamp approach. (**A**–**C**) Sample traces of capsaicin (50 nM)-activated current time courses in TRPV1-HEK cells pretreated with either PBS, PEDHC (1 μg/mL, *n* = 10), or PGDHC (1 μg/mL, *n* = 10). The holding potential was −60 mV. The horizontal bars denote the times when the indicated compounds were present in the bath. The kinetics of capsaicin-induced current decays were variable and did not significantly differ between the tested groups. Neither PEDHC (1 μg/mL) nor PGDHC (1 μg/mL) induced any currents in TRPV1-HEK cells when each dihydroceramide was applied alone in the absence of capsaicin. (**D**,**E**) Comparison of the differences between the amplitudes of peak capsaicin (50 nM)-induced currents recorded in the presence of either PBS, PEDHC (1 μg/mL), or PGDHC (1 μg/mL) indicated with (2) in panels A–C and the currents recorded in the presence of only PBS, PEDHC (1 μg/mL), or PGDHC (1 μg/mL) at the time point indicated with (1) in panels A–C. The data were collected at a holding potential of −60 mV. Each filled black circle indicates the value for each tested cell. The filled red circles represent the mean difference values for each experimental group, and the vertical red lines are the error bars (SEM). The t-test was used to compare the data sets. Statistically significant differences were observed between the experimental groups. (**F**) Current–voltage relationships for capsaicin (50 nM)-induced currents in the presence of either PBS or PEDHC (1 μg/mL). The gray area indicates ± SEM. The solid lines are the means of current–voltage relationships (PBS, *n* = 8; PEDHC, *n* = 10). (**G**) Current–voltage relationships for capsaicin (50 nM)-induced currents in the presence of either PBS or PGDHC (1 μg/mL). The gray area indicates ± SEM. The solid lines are the means of current–voltage relationships (PBS, *n* = 10; PGDHC, *n* = 10). (**H**) The voltage waveform protocol used during the whole-cell voltage-clamp experiments.

To validate our fluorescence imaging measurements, we also determined the ability of dihydroceramides to modulate the capsaicin-elicited currents in TRPV1-HEK cells using the whole-cell voltage-clamp approach. TRPV1-HEK cells were voltage-clamped at −60 mV, and capsaicin-induced currents were recorded (Figure 6A–C). Figure 6B,C show that adding either 1 μg/mL PEDHC or 1 μg/mL PGDHC alone to TRPV1-HEK cells in the recording chamber did not elicit any significant currents in TRPV1-HEK cells. However, pretreatment with either 1 μg/mL PEDHC or 1 μg/mL PGDHC significantly potentiated the capsaicin-stimulated currents in TRPV1-HEK cells held at −60 mV, compared to those in control TRPV1-HEK cells pretreated with PBS (Figure 6D–G). Conversely, at +100 mV, the capsaicin-elicited currents were neither significantly potentiated by 1 μg/mL PEDHC pretreatment, compared to PBS-treated TRPV1-HEK cells (Figure 6F, PEDHC, 381.42 ± 34.86 *n* = 10; PBS, 354.35 ± 81.39, *n* = 8, *p* = 0.746), nor by 1 μg/mL PGDHC pretreatment, compared to PBS-treated TRPV1-HEK cells (Figure 6G, PGDHC, 464.32 ± 111.73, *n* = 10; PBS, 309.05 ± 82.34 pA/pF, *n* = 10, *p* = 0.212).

**Figure 7:**
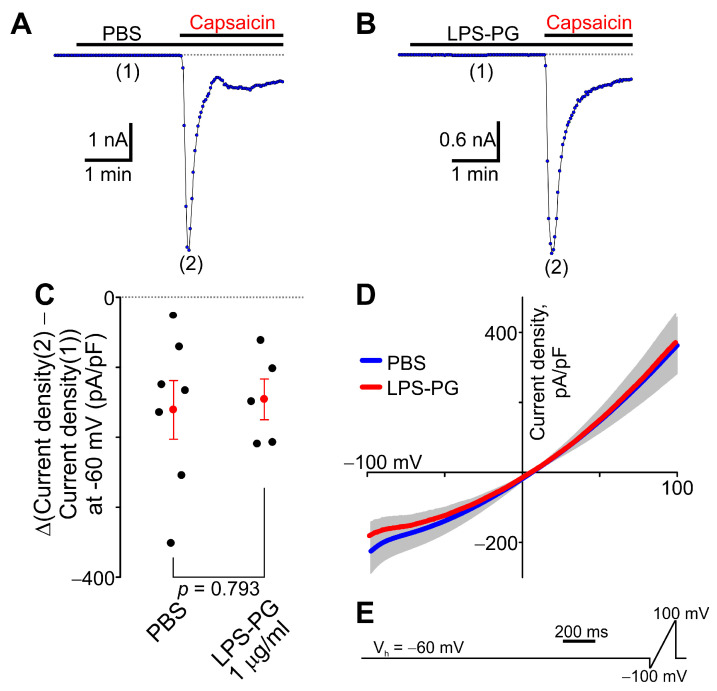
Effect of LPS-PG on capsaicin-activated currents in TRPV1-HEK cells. (**A**,**B**) Sample traces of capsaicin (50 nM)-activated current time courses in TRPV1-HEK cells pretreated with either PBS (*n* = 7) or LPS-PG (1 μg/mL, *n* = 5). Whole-cell voltage-clamp recordings are shown. The holding potential was −60 mV. The horizontal bars denote the times when the indicated compounds were present in the bath. LPS-PG (1 μg/mL) did not induce any currents in TRPV1-HEK cells when it was added to the recording chamber alone. (**C**) Comparison of the differences between peak capsaicin (50 nM)-induced current amplitudes recorded in the presence of either PBS or LPS-PG (1 μg/mL) indicated with (2) in panels A–B and the currents recorded in the presence of PBS or LPS-PG (1 μg/mL) alone at a time point indicated with (1) in panels A–B. The data were collected at a holding potential of −60 mV. Each filled black circle indicates the difference value for each tested cell. The filled red circle represents the mean value for each experimental group, and the vertical red lines are the error bars (SEM). The t-test was used to compare the data sets. There was no statistically significant difference between the two experimental groups. (**D**) Current–voltage relationships for capsaicin (50 nM)-induced currents in the presence of either PBS or LPS-PG (1 μg/mL). The gray area indicates ± SEM. The solid lines are the means of current–voltage relationships (PBS, *n* = 7; LPS-PG, *n* = 5). (**E**) the voltage waveform protocol used during the whole-cell voltage-clamp experiments.

We next investigated whether LPS-PG would have any effect on capsaicin-induced currents in TRPV1-HEK cells. No significant difference was observed between the LPS-PG (1 μg/mL) and PBS groups during our electrophysiological experiments (Figure 7). The higher concentration of LPS-PG was not tested due to biosafety concerns.

## 3. Discussion

We report here that *Bacteroidetes P. gingivalis*-derived PEDHC and PGDHC [16,18] but not LPS-PG can sensitize TRPV1 channels in a mammalian-expression HEK cell model. All three bacterially derived components, PEDHC, PGDHC, and LPS-PG, can potentially activate both the CD14/TLR2 and CD14/MD2/TLR4 complexes [14,19,20]. Specifically, LPS can activate TLR4 via its accessory molecules, CD14 and MD2. MD2 is not required for the functional engagement of TLR2 [20]. In this study, we used ultrapure LPS-PG that only signals through CD14/MD2/TLR4 [18]. However, HEK cells do not express endogenous CD14/MD2/TLR4 and CD14/TLR2 complexes [21,22]. Therefore, we employed this heterologous mammalian expression model to determine whether there is a CD14/TLR2- or a CD14/MD2/TLR4-independent pathway underlying the possible effects of the bacterial components on the human TRPV1 channel heterologously expressed in HEK cells.

Cell-permeant PEDHC and PGDHC are structural homologues to mammalian dihydroceramides and sphingomyelin [14,16]. It was previously reported that neither ceramide nor sphingosine influenced TRPV1 function in trigeminal neurons or in TRPV1-expressing CHO cells [23]. However, the disruption of lipid rafts containing sphingomyelin, by depleting cholesterol or cleaving sphingomyelin with sphingomyelinase, inhibited TRPV1 activity in trigeminal neurons and TRPV1-CHO cells, indicating the importance of lipid rafts for TRPV1 function [23]. Those findings also suggested that sphingomyelin may have a permissive role for TRPV1 activation. Thus, our data are consistent with the role of sphingomyelin-like molecule on TRPV1 activity.

It was reported that there is a resident phosphatidylinositol lipid in the vanilloid binding site of the TRPV1 protein and that vanilloid agonists, such as capsaicin, need to displace the endogenous lipid to activate the channel [24]. The C-terminal sites for phosphoinositide 4,5-bisphosphate binding were also identified in the TRPV1 protein [25,26]. The molecular structure of at least PEDHC has some resemblance to phosphatidylinositol lipids (Figure 1). Thus, PEDHC may potentially bind to the TRPV1 protein within or near the capsaicin binding site or its C-terminus. Conversely, it has been proposed that phosphoinositides can modulate TRPV1 function indirectly via a membrane protein, Pirt [27]. Currently, it is unclear whether PEDHC or PGDHC directly interact with the TRPV1 protein or whether these dihydroceramides’ effects are due to an indirect action via some other proteins or intracellular signaling pathways contributing to modulating TRPV1 function.

We found that LPS-PG pretreatment did not significantly affect TRPV1-mediated Ca^2+^ influx or capsaicin-elicited inward currents in TRPV1-HEK cells. Likely, this is because TRPV1-HEK cells express neither CD14/MD2/TLR4 nor CD14/TLR2. Sattler et al. 2021 reported that a 6 h pretreatment with LPS (1 μg/mL) promoted TRPV1 channel internalization and decreased TRPV1 activity in human-induced pluripotent stem-cell-derived cardiomyocytes [28]. We used only 1–3 min LPS-PG pretreatments, so it remains to be determined whether a longer LPS-PG treatment could cause TRPV1 internalization in HEK cells.

In conclusion, our data indicated that both PEDHC and PGDHC may contribute to the sensitization of TRPV1 channels in a concentration-dependent manner, whereas LPS-PG did not cause significant TRPV1 sensitization. Neither PEDHC nor PGDHC alone activated TRPV1. PEDHC appears to be a less potent TRPV1 sensitizer, compared to PGDHC, because PEDHC was effective at a higher concentration of 10 μg/mL in our fluorescence imaging experiments. Conversely, PGDHC efficiently sensitized TRPV1 at a lower concentration of 1 μg/mL in our fluorescence imaging experiments. Possibly, PEDHC exhibited a lower affinity to its cellular target in TRPV1-HEK cells. However, further experiments are needed to support or refute this hypothesis. Further in vivo experiments are also needed to determine whether any of the shed bacterial products affect pain sensitivity in infected wounds or during pulpitis. Clinical observations demonstrate that there is a significant burden of human wounds worldwide [29]. Wounds may arise from surgical procedures, diabetic foot ulcers, burns, pressure ulcers, animal bites, domestic violence, fall-related injuries, combat-related injuries, sports-related injuries, etc. [5]. Pain is a common clinical problem in patients with various types of wounds, affecting their quality of life. It is important to further understand the mechanisms underlying increased pain sensitivity in infected wounds.

## 4. Materials and Methods

### 4.1. Cell Culture and Transfection

HEK cells were purchased at American Type Culture Collection (Manassas, VA, USA). The cells were cultured in Eagle’s minimum essential medium supplemented with 10% fetal bovine serum. Cells were transfected with the human TRPV1 cDNA (OriGene Technologies, Inc. Rockville, MD, USA) using Lipofectamine 3000 (Thermo Fisher Scientific, Waltham, MA, USA), in accordance with the recommendations of the manufacturer. To facilitate the identification of transfected cells, TRPV1 cDNA was co-transfected with Green Fluorescent Protein (GFP) cDNA. The transfected cells were plated on 35 mm plastic Petri dishes and cultured for 3 days before the transfection. After transfection, cells were split and plated on 25 mm glass coverslips and cultured for additional 48 h.

### 4.2. Fluorescence Imaging

Cells were loaded with the Fura-2 fluorescent dye for ratiometric intracellular Ca^2+^ imaging experiments as described elsewhere [30,31]. The Ca^2+^ imaging experiments were performed using a Till Photonics single-cell fluorescence imaging system integrated with an inverted Zeiss Axio Vert A1 microscope (Zeiss, Oberkochen, Germany) with a custom set of filter cubes to observe Fura-2 and GFP fluorescences. The fluorescence of Fura-2 was alternatively excited at wavelengths of 340 nm and 380 nm. Fluorescent light was collected using an Andor DU885 charge-coupled device camera (Andor Technology PLC, South Windsor, CT, USA) after passing through a long-pass 510 nm filter. The fluorescence-intensity ratio (F_340_/F_380_) was calculated and plotted versus time. TRPV1 channels were activated with 50 nM capsaicin. A syringe pump-based flow-through system was used to superfuse the transfected cells (TRPV1-HEK cells) with the experimental solutions at a rate of 1 mL/min. The extracellular solution contained (in mM) 145 NaCl, 1 CaCl_2_, 2 MgCl_2_, 2.5 KCl, 10 HEPES, and 5.5 Glucose (pH 7.2). Cells that had a resting F_340_/F_380_ ratio greater than 1 or did not show a reversible capsaicin response were not included in the statistical analysis. Cells were pretreated with the bacterial membrane components for 3 min before TRPV1 channels were activated with capsaicin.

### 4.3. Electrophysiological Recordings

Electrophysiological experiments were performed as described elsewhere [31,32,33]. Briefly, HEK cells were plated at a low density on round 25 mm glass coverslips. The experiments were performed 48 h after transfection of the cells with a mixture of TRPV1 and GFP cDNA (4 μg and 0.5 μg, respectively). An Axopatch 200B amplifier and Digidata 1400 digitizer (Molecular Devices, San Jose, CA, USA) were employed to record the currents in TRPV1-HEK cells using the whole-cell voltage-clamp mode. Series resistance compensation was set to 50–70%. The holding potential was set to −60 mV. Then, 150 ms voltage ramps from −100 to +100 mV were applied every 2 s. The data were filtered at 3 kHz. The reversal potentials of the capsaicin-induced currents amounted to 6.10 ± 0.61 mV in the presence of PBS (*n* = 10), 5.98 ± 0.79 mV in the presence of PEDHC (*n* = 10), 5.9 ± 0.62 mV in the presence of PGDHC (*n* = 10), and 5.94 ± 0.71 mV in the presence of LPS-PG (*n* = 5), with no significant difference being found in the mean values of the reversal potentials among the experimental groups (*p* = 0.997). The extracellular solution contained (in mM) 145 NaCl, 2.5 KCl, 1 CaCl_2_, 1 MgCl_2_, 10 HEPES, and 5.5 glucose (pH 7.2 adjusted with NaOH). The pipette solution contained (in mM) 140 CsMeSO_3_, 10 CsCl, 2 MgCl_2_, 0.5 EGTA, and 10 HEPES (pH 7.2 adjusted with Trizma base) for PEDHC experiments and 125 CsMeSO3, 3.77 CaCl2, 2 MgCl2, 10 EGTA, and 10 HEPES (pH 7.2 adjusted with Trizma base) for PGDHC and LPS-PG experiments. The flow in the recording chamber was maintained only during applications of experimental solutions. A 2–3 mL volume of each experimental solution was added to the 0.25 mL bath at a rate of 5–10 mL/min to reach the final concentration for each compound. Cells were pretreated with the bacterial membrane components for 1.5–3 min before TRPV1 channels were activated with capsaicin. No leak subtraction was done. Cells with leak currents greater than 100 pA were not included in the statistical analysis. pCLAMP 10 software was used for data acquisition and analyses. The average cell capacitance was 19.92 ± 1.54 pF (*n* = 53). The experiments were performed at 22–25 °C.

### 4.4. Materials

PGDHC and PEDHC were isolated from *P. gingivalis* (ATTC, strain #33277) and prepared for in vitro studies as described elsewhere [3]. PEDHC, PGDHC, and LPS (from *P. gingivalis*, Catalog # tlrl-ppglps, InVivoGen, San Diego, CA, USA) were dissolved in phosphate-buffered saline (PBS) containing Ca^2+^ and Mg^2+^ and were added to the standard extracellular solution just before the experiment, following a 30 s sonication for reconstituting the bacterial components in the water-based solution. Capsaicin was dissolved in DMSO and then diluted in the extracellular solution to a concentration of 50 nM.

### 4.5. Statistical Analysis

The statistical analysis was performed using SigmaPlot 12.5 software. To determine whether there was a statistically significant difference between the tested groups, we used the Kruskal–Wallis one-way ANOVA on ranks test, followed by the Dunn’s post hoc multiple comparisons versus control group test. The t-test was used to compare electrophysiological data sets. The significance level was set to P < 0.05.

## Data Availability

Not applicable.
